# Transcriptomic insights into loperamide-induced gastric dysmotility: RNA-seq analysis of a mouse model

**DOI:** 10.3389/fphys.2026.1924065

**Published:** 2026-10-07

**Authors:** Chunggeon Lee, Seung-Ju Hwang, Young-Su Yang, Bokyung Kim, Dong-Seon Kim, Daeui Park, Chang-Woo Song, Chang-Gue Son

**Affiliations:** 1 Center for National Toxicology AI and Data, Korea Institute of Toxicology, Daejeon, Republic of Korea; 2 School of Korea Institute of Toxicology, University of Science and Technology, Daejeon, Republic of Korea; 3 Institute of Bioscience & Integrative Medicine, Daejeon University, Daejeon, Republic of Korea; 4 Division of Jeonbuk Advanced Bio Research, Korea Institute of Toxicology, Jeongeup, Republic of Korea; 5 Translational Toxicology Technical Research Team, Division of Jeonbuk Advanced Bio Research, Korea Institute of Toxicology, Jeongeup, Republic of Korea; 6 Korean Medicine Science Research Division, Korea Institute of Oriental Medicine, Daejeon, Republic of Korea

**Keywords:** functional dyspepsia, gastrointestinal motility, loperamide, RNA sequencing, synapse-related pathway

## Abstract

**Background:**

Loperamide, a μ-opioid receptor agonist that slows gastrointestinal transit, is widely used as an antidiarrheal agent and an experimental model of gastric dysmotility and functional dyspepsia (FD). However, transcriptomic alterations associated with loperamide-induced delayed gastric emptying remain poorly characterized. This study aimed to investigate gastric gene-expression profiles associated with loperamide-induced gastric dysmotility using RNA sequencing.

**Methods:**

Male BALB/c mice were intraperitoneally injected with loperamide after 20 h of fasting. Delayed gastric emptying was assessed in one cohort, while gastric antrum tissues from a separate cohort were collected for RNA sequencing. Differentially expressed genes (DEGs) were identified using fold change ≥1.5 and false discovery rate (FDR) <0.01. Gene set enrichment analysis (GSEA) and pathway-network analysis were conducted to characterize biological pathways associated with loperamide treatment.

**Results:**

Loperamide significantly delayed gastric emptying, as demonstrated by increased gastric retention of phenol red. Transcriptomic analysis identified 1,356 DEGs, including 553 upregulated and 803 downregulated genes. GSEA revealed enrichment of inflammatory response-related pathways among upregulated genes, whereas synaptic transmission- and calcium signaling-related pathways were predominantly downregulated. Representative genes involved in cholinergic signaling (*Chrm2* and *Chrna9*), glutamatergic signaling (*Grm7*), and calcium-channel function (*Cacna1a*) showed reduced expression, while *Il33* was increased.

**Conclusion:**

This study provides transcriptomic insights into loperamide-induced gastric dysmotility. The findings suggest coordinated downregulation of genes associated with neuronal/synaptic signaling together with enrichment of inflammatory response-related transcriptional pathways in the gastric antrum, providing a molecular framework for this widely used experimental model.

## 1 Introduction

Gastrointestinal (GI) motility is crucial for regulating digestive processes by coordinating the movement of ingested material through different segments of the digestive tract (Camilleri, 2023). Given the vital importance of digestive function, disruption in GI motility can result in various pathological conditions, particularly functional dyspepsia (FD), gastroparesis, constipation, and diarrhea associated with irritable bowel syndrome (IBS) (Kusano et al., 2014).

GI motility is mainly regulated by both intrinsic and extrinsic nervous systems, which consist of sympathetic and parasympathetic inputs (Spencer and Hu, 2020). The intrinsic nervous system, referred to as the enteric nervous system (ENS), coordinates digestion, secretion, and motility through a complex neuronal network, while the autonomic nervous system exerts control over GI blood flow, gastric and intestinal tone, and motility (Wood, 2016). Accordingly, many prokinetic drugs that target the regulation of GI motility involve the mechanical actions of these nervous systems. For example, 5-hydroxytryptamine receptor 4 (5-HT_4_R) agonists such as mosapride citrate activate enteric neurons within the myenteric and submucosal plexuses of the ENS along with an indirect affecting on the parasympathetic nerve, resulting in the treatment of the delayed gastric emptying in FD (Tack et al., 2019).

On the contrary, μ-opioid receptor agonists such as morphine and loperamide slow down intestinal transit thus managing diarrhea, which inhibits the release of excitatory neurotransmitters such as acetylcholine on neurons within ENS and parasympathetic outflow (Wu and Juurlink, 2017). Meanwhile, approximately 40% of chronic opioid users experience GI dysmotility (Pappagallo, 2001). Loperamide has become further prescribed by 91% from 2010 to 2015, and its frequent use leads to adverse effects like constipation, stomach discomfort, vomiting, and abdominal pain (Vakkalanka et al., 2017; Malinky et al., 2021). The loperamide treatment has also been used for animal models of FD, based on that its overdosage or prolonged treatment-related delay in GI motility can be observed in encompassing rodents and zebrafish (Hwang et al., 2021). While much is understood about how loperamide delays gastric emptying, some aspects remain unclear, including the molecular mechanism and the exact intracellular signaling pathways involved in these processes.

RNA sequencing (RNA-seq) technology offers a new strategy to comprehensively study the transcriptional landscape of key genes involved in conditioned tissues and cells (Nagalakshmi et al., 2010). Recent advances in omics technologies have enabled the elucidation of previously unknown mechanisms of pharmacological agents and disease pathology, as well as to identification of novel biomarkers. Although loperamide is widely used clinically and its effects on gastrointestinal motility are well established, the transcriptomic alterations associated with loperamide-induced gastric dysmotility remain poorly characterized, particularly in gastric tissue. Previous transcriptomic studies of loperamide-induced gastrointestinal dysfunction have primarily focused on intestinal or colonic tissues, leaving the molecular characteristics of the gastric response less well defined. Therefore, the present study aimed to characterize the gastric transcriptomic alterations associated with loperamide-induced delayed gastric emptying using RNA-seq analysis of the gastric antrum. Our analysis identified enrichment of inflammatory response-related pathways and downregulation of synaptic transmission- and calcium signaling-related pathways following loperamide treatment.

## 2 Materials and methods

### 2.1 Chemicals and reagents

The following reagents and chemicals were purchased from Sigma Aldrich (MO, United States): loperamide hydrochloride, phenol red, sodium carboxymethyl cellulose (CMC-Na), sodium hydroxide, trichloroacetic acid (TCA). Other reagents and chemicals were obtained from the following manufacturers: arabic gum (JUNSEI, Tokyo, Japan).

### 2.2 Animals and experimental design

A total of 22 Balb/C male mice (6 weeks old; 19–21 g) were purchased from Daehanbio-link (Eumseong-gun, Chung-buk, Korea). These animals were maintained at room temperature (22 ± 2 °C) and 60 ± 5% relative humidity under a 12 h light: 12 h dark cycle. The mice were given free access to a commercial pellet diet (Daehanbio-link) and tap water. After 7 days of acclimatization, the mice were randomly divided into two groups (n = 11/group): the normal and loperamide groups. From each group, 5 mice were used to measure gastric emptying and the remaining 6 mice were used to obtain gastric antrum tissue for RNA sequencing analysis. Before the evaluation day, the mice were fasted for 20 h. After 20 h of fasting, the mice were subjected to one of two separate experimental protocols: (1) assessment of gastric emptying (n = 5/group) or (2) gastric antrum collection for bulk RNA sequencing (n = 6/group). The detailed procedures and timing for each experimental protocol are described below. Experimental design was shown in **Figure 1A**. The protocol was approved by the Institutional Animal Care and Use Committee of Daejeon University (Daejeon, Korea; Approval No. DJUARB 2021-013) and was conducted in accordance with the Guide for the Care and Use of Laboratory Animals, published by the National Institutes of Health (NIH, MD, United States).

**Figure 1:**
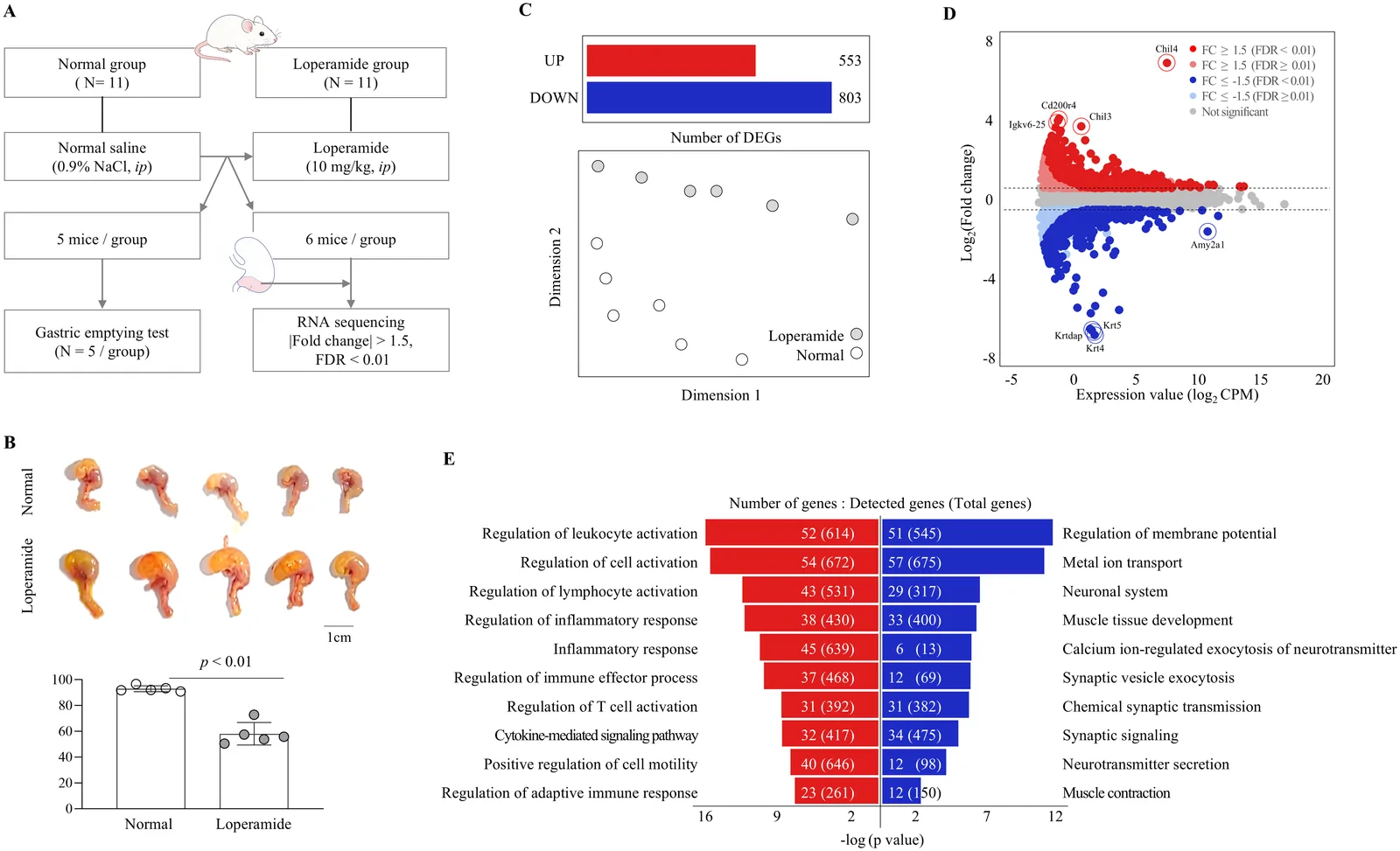
Loperamide-induced delayed gastric emptying and associated transcriptomic changes in gastric antrum of mouse. **(A)** Balb/C, male mice (N = 11/group) were injected with normal saline or loperamide (10 mg/kg). Stomach tissues were isolated from each group (N = 6/group) and total RNA were extracted from tissues, then analyzed to determine DEGs. **(B)** Gastric emptying was determined by pictures of phenol red-retained stomach and concentration of retained phenol red in stomach. The DEGs between the normal group and the loperamide-treated group were identified. **(C)** In the DEGs with a fold change ≥ 1.5 and FDR < 0.01, the number of upregulated genes was 553, and the number of downregulated genes was 803. The MDS plot was conducted using 1,356 genes, after filtering out genes likely to be noise from the RNA-seq data, revealing a clear separation between the normal group and the loperamide-treated group. **(D)** The MA plot indicates the distribution of RNA-seq data. Each dot represents upregulated genes (red), downregulated genes (blue), and not significant genes (gray). The X axis represents the expression values of genes using log_2_ CPM, and the Y axis represents the expression level of genes using log_2_ (Fold change). **(E)** The 10 biological pathways associated with DEGs were visualized in the bar plot. The red bar plot (left) represents biological pathways associated with upregulated genes, while the blue bar plot (right) represents biological pathways associated with downregulated genes. In the bar plot, the number of genes shows the DEGs belonging to the pathways and the total gene sets of the pathways.

For euthanasia, mice were euthanized in a CO_2_ chamber (Jeungdo Bio & Plant, Seoul, South Korea) by gradual-fill method at an inflow rate of approximately 30% of chamber volume/minute. Animals were exposed to CO_2_ for 5 minutes and euthanasia was confirmed by cessation of spontaneous respiration and absence of reflexes prior to tissue collection.

### 2.3 Determination of gastric emptying using phenol red

The mice were fasted for 20 h and given free access to tap water. Mice of loperamide group were intraperitoneally injected with loperamide hydrochloride (10 mg/kg, dissolved in normal saline). And mice of the normal group were injected with normal saline (10 ml/kg). The dose of loperamide (10 mg/kg) was selected based on our previous experimental model and published protocols demonstrating a robust and reproducible delay in gastric emptying (Hwang et al., 2021). After 30 min, all the mice were orally administered phenol red solution (500 μL/mouse). The Phenol red solution consisted of 1.5% Na-CMC and 0.05% phenol red in distilled water. Thirty minutes after phenol red treatment, the mice were euthanized in a CO_2_ chamber (Jeungdo Bio and Plant, Seoul, Korea), and then, the stomachs were immediately removed. In the experiment to determine gastric emptying, the optimal volume of phenol red solution and time point at which approximately 60% delayed gastric emptying was observed were established by our previous study (Hwang et al., 2021) and other protocol (Asano et al., 2016). To measure the absorbance of the phenol red retained in the stomach, stomach samples were homogenized in 5 mL of 0.1 N sodium hydroxide solution and 0.5 mL of 20% TCA. The homogenates were centrifuged at 3,000 rpm for 20 min, and then, 1 mL of supernatant was added to 4 mL of 0.5 N sodium hydroxide. Finally, the absorbance of these pink-colored solutions was determined at 560 nm by using a spectrophotometer. The gastric emptying rates were calculated according to the following formula: Gastric emptying (%) = (1 − X/Y) × 100. X: Absorbance of stomach-retained phenol red, Y: Absorbance of naïve phenol red mixed with sodium hydroxide.

### 2.4 Sample preparation for transcriptomic analysis

For transcriptomic analysis, mice were fasted for 20 h with free access to water and then received a single intraperitoneal injection of loperamide hydrochloride (10 mg/kg) or normal saline (10 mL/kg). Gastric antrum tissues were collected 1 h after loperamide or vehicle administration. Unlike the animals used for the gastric emptying test, the mice assigned to transcriptomic analysis did not receive phenol red solution.

Total RNA was isolated from gastric antrum tissues obtained from the normal group (N = 6) and loperamide-treated group (N = 6) (**Figure 1A**). The total RNA sequencing library was prepared using TruSeq Stranded Total RNA Library Prep kit with Ribo-zero mouse (Illumina, CA, United States). The first strand cDNA is synthesized using reverse transcriptase and random primers, and the second strand cDNA is synthesized using DNA polymerase I and RNase H. The final cDNA libraries were created by adding a single ‘A’ base and adapter ligation and then purified and enriched with PCR. The quality of these libraries was assessed by automated electrophoresis (Tapestation, Agilent, United States). 101-bp paired-end reads were generated by using NovaSeq6000 (Illumina, CA, United States) for RNA sequencing. For real-time PCR validation, total RNA obtained from six animals per group was pooled by combining RNA from two individual mice, generating three pooled biological samples per group (n = 3 pooled samples/group). Pooling was performed to reduce inter-individual variability and to ensure sufficient RNA quantity for downstream analyses. Real-time PCR was performed using the same RNA samples used for RNA-seq validation.

### 2.5 Genome mapping and identification of differentially expressed genes

The 101-bp paired-end reads were aligned to the Genome Reference Consortium Mouse Build 39 (GRCm39) using Spliced Transcripts Alignments to a Reference (STAR) version 2.7.10a (Dobin et al., 2013). The gene expression levels were calculated by the featureCounts in Subread 2.0.3 which was used to process the resulting binary alignment and map (BAM) files (Liao et al., 2014). The Differentially Expressed Genes (DEGs) between the loperamide-treated group and the normal group were identified as those with a fold change ≥ 1.5 and FDR < 0.01, using edge R in a Bioconductor package (Robinson et al., 2010). Total DEGs were described in **Supplementary Tables 1**, **2**.

### 2.6 Gene Set Enrichment Analysis

Gene Set Enrichment Analysis (GSEA) is a useful method for identifying gene sets that are enriched in biological pathways. For GSEA, DEGs were analyzed using the Metascape tools (Zhou et al., 2019). Significant pathways related to DEGs with *p value* under 0.01 were identified. The clustering of biological pathways was presented as a pathway-pathway interaction network using Cytoscape (v3.10.3) (Shannon et al., 2003). In the network analysis, the nodes represent each pathway that includes gene sets, and the edges describe the interactions between pathways using pairwise similarity scores. The pairwise similarities of pathways were calculated using the Kappa score, which measures the similarity based on the ratio of the number of intersecting genes to the number of genes in the union of the two pathways. The total pathways and contained DEGs were described in more detail in **Supplementary Table 3**.

### 2.7 Heatmap and clustering analysis

The multidimensional scaling (MDS) plot is a plot of the distances between the samples in the loperamide-treated group and the normal group. The distance between the two samples was calculated by Euclidean distance based on gene expression value. In addition, heatmaps and boxplots were utilized to illustrate the expression values of DEGs within a specific biological pathway, visualizations were generated based on the DEGs. Heatmap images were generated using the pheatmap R package (https://rdocumentation.org/packages/pheatmap/versions/1.0.12). In the heatmap, expression values were scaled using the Z-score formula. The boxplot from the ggplot R package was used to compare the gene expression levels between the two groups (https://ggplot2.tidyverse.org/index.html), and *p values* were calculated using a unpaired t-test.

### 2.8 Real-time PCR analysis

The High-Capacity cDNA Reverse Transcription Kit (4368814, Thermo Fisher Scientific, USA) was used to synthesize cDNA from 2 μg of total RNA. The SYBR Green PCR Master Mix (Applied Biosystems, USA) was used to conduct real-time PCR. The primers utilized for amplifying genes, including Chrm2, Chrna9, Grm7, Cacna1a, Il33, and Gapdh, are detailed in **Supplementary Table 4**. The IQ5 PCR Thermal Cycler (Bio-Rad, USA) was utilized to analyze gene expression data.

### 2.9 Statistical analysis

The gastric emptying and real-time PCR data are expressed as the mean ± standard deviation (SD) or fold changes in means. Statistical significance was determined by using the unpaired Student’s t-test. In all analyses, *p value* < 0.05 was considered to indicate statistical significance.

## 3 Results

### 3.1 Delayed gastric emptying by loperamide

As expected, loperamide injection delayed the passage of phenol red, leading to the fullness of the stomach compared to the normal group (*p* < 0.01), as observed by the naked eye, and quantification of the amount of phenol red retained in the stomach (**Figure 1B**).

### 3.2 Sample clustering and DEGs between loperamide group and normal group

A total of 1,356 DEGs were identified (553 upregulated and 803 downregulated genes), which clustered well into two groups in MDS analysis (**Figure 1C**). As shown in the MA plot, 76 genes and 131 genes were over 4-fold up- and down-regulated in loperamide-treated gastric tissues compared to the normal sample (**Figure 1D**). The 10 up- and down-regulated genes were listed in **Table 1**.

**Table 1:** The top 10 up- and down-regulated genes.

Genes	Description	Fold change^*^	FDR^**^	Expression value^***^
A. The top 10 up-regulated genes				
Chil4	chitinase-like 4	163.00	8.21.E-12	178.61
Cd200r4	CD200 receptor 4	20.29	5.33.E-09	0.32
Chil3	chitinase-like 3	15.23	7.21.E-07	1.19
Igkv6-25	immunoglobulin kappa chain variable 6-25	14.59	5.95.E-05	0.26
Mrgpra9	MAS-related GPR, member A9	12.83	1.32.E-10	0.35
Zap70	zeta-chain (TCR) associated protein kinase	12.82	2.70.E-10	0.35
Bdnf	brain derived neurotrophic factor	12.17	1.27.E-08	0.34
Pkd1l2	polycystic kidney disease 1 like 2	10.74	5.21.E-06	0.21
Ighv1-42	immunoglobulin heavy variable V1-42	10.09	8.59.E-04	0.30
Foxe1	forkhead box E1	9.39	1.59.E-06	0.28
B. The top 10 down-regulated genes				
Krt4	keratin 4	-163.04	7.10.E-12	2.55
Krtdap	keratinocyte differentiation associated protein	-138.13	1.14.E-12	2.18
Krt5	keratin 5	-130.60	2.14.E-14	2.01
Ltf	lactotransferrin	-72.85	2.91.E-10	2.04
Krt1	keratin 1	-64.21	5.40.E-12	10.86
Serpinb12	serine (or cysteine) peptidase inhibitor, clade B (ovalbumin), member 12	-58.81	3.96.E-10	0.94
Krt14	keratin 14	-55.29	1.80.E-10	2.71
Rptn	repetin	-33.81	2.64.E-09	4.14
Fmr1nb	Fmr1 neighbor	-30.90	5.69.E-08	0.75
Krt16	keratin 16	-26.57	7.30.E-07	0.83

*Fold change was obtained by discarding the third decimal place

**FDR was obtained by rounding to the third decimal place

***Expression value is CPM (Counts per million).

### 3.3 Expression changes of Gene Set Enrichment by loperamide

GSEA was employed to discern the biological pathways affected by loperamide treatment, and the top 10 up- or down-regulated pathways were selected. In particular, the “Regulation of inflammatory response (38 genes, *p value* = 2.08E-12)” and “Inflammatory response (45 genes, *p value* = 4.55E-11)” were notably upregulated, while “Neuronal system (29 genes, *p value* = 4.12E-07)” and “Chemical synaptic transmission (31 genes, *p value* = 2.18E-06)” pathways were downregulated (**Figure 1E**).

### 3.4 Analysis for synaptic pathway-related genes down-regulated by loperamide

Regarding the genes belonging to the synaptic pathway, 31 genes were significantly downregulated by loperamide, which specifically are linked to the function of acetylcholine receptors, glutamate receptors, and calcium channels (**Figure 2A**).

**Figure 2:**
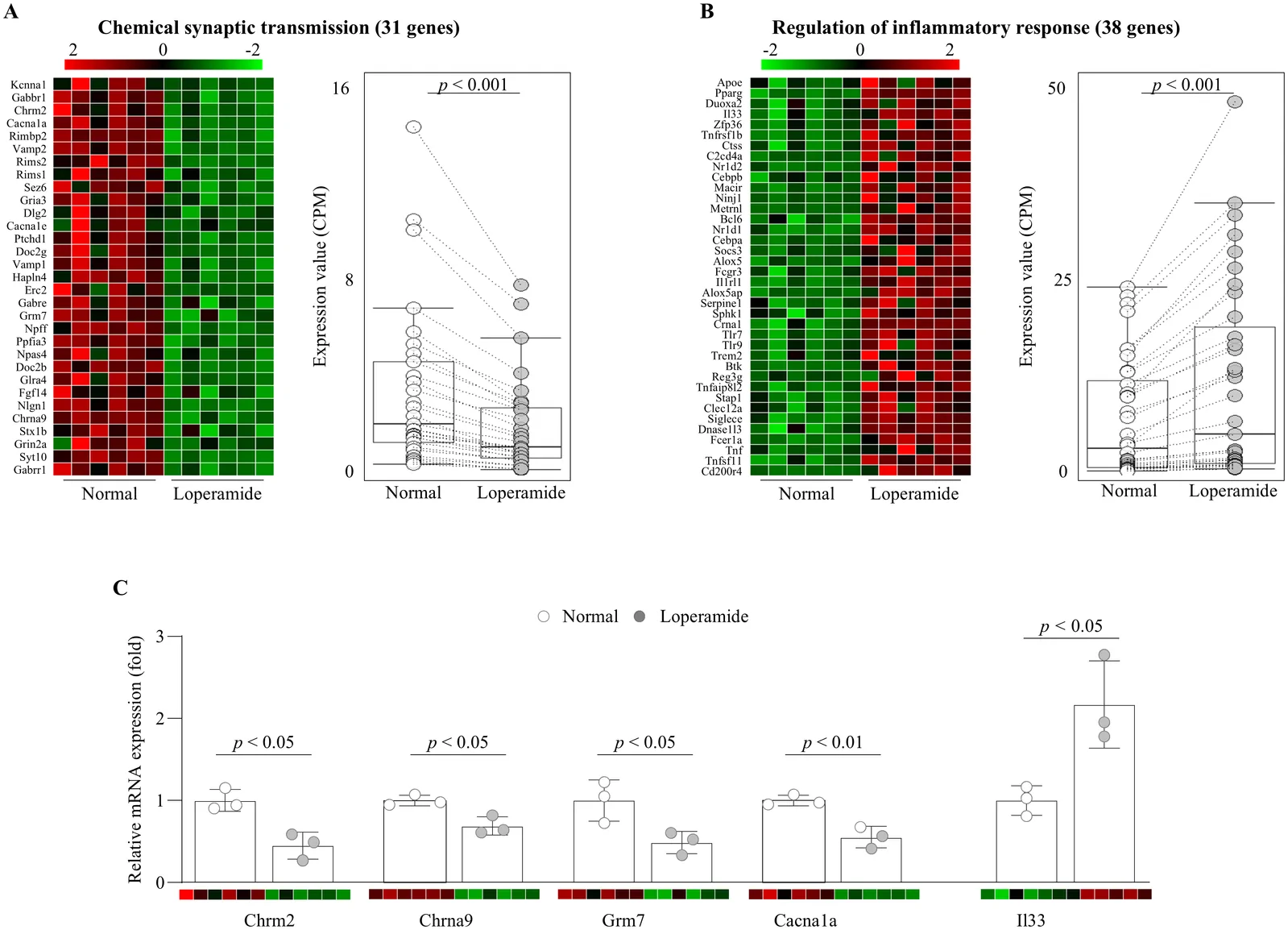
Analysis of synaptic and inflammatory response-related genes. The expression patterns of genes, including each biological pathway, were visualized using heatmaps and boxplots. The heatmaps and boxplots were generated using Counts Per Million (CPM) values. In the heatmap, the expression values of genes are scaled using Z-score formula. In the boxplot, the dots depict the average expression values of each gene in each group, and the presence of shared genes between two conditions is indicated by the use of dashed lines. The *p value* in the boxplot was calculated using unpaired Student's t-tests. **(A)** The genes belonging to the “Chemical synaptic transmission” were significantly downregulated after treatment with loperamide. **(B)** The genes belonging to the “Regulation of Inflammatory response” showed a significant upregulation after treatment with loperamide. **(C)** Representative genes, including Chrm2, Chrna9, Grm7, Cacna1a, and Il33, were validated by real-time PCR using the same RNA samples employed for RNA-seq analysis. RNA obtained from six mice per group was pooled in pairs, resulting in three pooled biological samples per group (n = 3). Chrm2, cholinergic receptor muscarinic 2; Chrna9, cholinergic receptor nicotinic alpha 9 subunit; Grm7, glutamate metabotropic receptor 7; Cacna1a, calcium voltage-gated channel subunit alpha1 A; Il33, interleukin 33.

For example, acetylcholine-related genes included cholinergic receptor nicotinic alpha polypeptide 9 (Chrna9, 2.5-fold) and cholinergic receptor muscarinic 2 (Chrm2, 1.9-fold), whereas two genes, glutamate receptor, ionotropic, NMDA2A (epsilon 1) (Grin2a, 5.7-fold), glutamate receptor, metabotropic 7 (Grm7, 1.6-fold), and glutamate receptor, ionotropic, AMPA3 (alpha 3) (Gria3, 1.6-fold). In addition, calcium-related genes included calcium channel, voltage-dependent, gamma subunit 5 (Cacng5, 5.2-fold), calcium channel, voltage-dependent, R type, alpha 1E subunit (Cacna1e, 1.7-fold), and calcium voltage-gated channel subunit alpha 1A (Cacna1a, 2.1-fold) were significantly downregulated.

When we confirmed the feature of gene expression using real-time PCR, those results were the same with RNA Seq data for Chrm2 (2.2-fold, *p* < 0.05), Chrna9 (1.5-fold, *p* < 0.05), Grm7 (2.0-fold, *p* < 0.05), and Cacna1a (1.5-fold, *p* < 0.01), respectively (**Figure 2C**).

### 3.5 Analysis for inflammatory response pathway-related genes up-regulated by loperamide

Regarding the genes belonging to the inflammatory response regulation pathway, loperamide treatment significantly upregulated the expression of 38 genes (**Figure 2B**). These genes included tumor necrosis factor (ligand) superfamily, member 11 (Tnfsf11, 5.2-fold), interleukin 1 receptor-like 1 (Il1rl1, 4.0-fold), tumor necrosis factor (tnf, 3.6-fold), and interleukin 33 (Il33, 2.4-fold). Using real-time PCR, we confirmed the upregulated Il33 expression level (2.2-fold, *p* < 0.05) (**Figure 2C**).

### 3.6 Network analysis-based explanation of loperamide’s actions

From the network analysis, we could find an overview of loperamide-induced changes in biological pathways in mouse stomach tissue. These results showed patterns of inhibition of synaptic transition, including calcium ion regulation and muscle contraction (**Figure 3A**), while the activation of gene sets related to inflammatory response regulation (**Figure 3B**).

**Figure 3:**
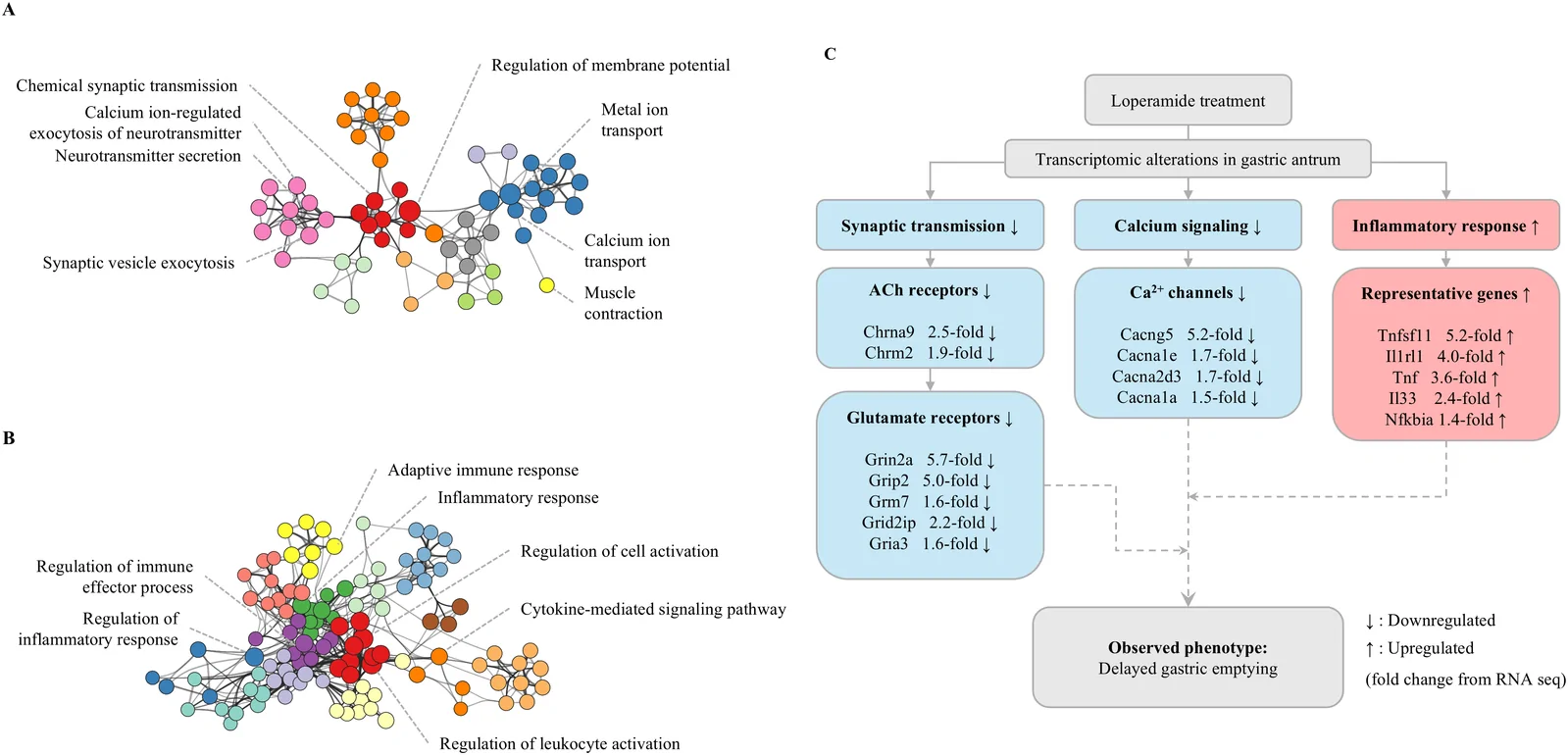
Network analysis of interconnected pathways and proposed transcriptomic framework of loperamide-induced gastric dysmotility. The network of biological pathways consists of nodes representing biological pathways, and edges representing similarities among these pathways. The size of nodes represents the number of genes in the pathways, while the color indicates the *p value*, and the similarities indicated by edges are based on the Kappa scores. **(A)** The network of biological pathways consisting of downregulated genes showed interactions among pathways related to synapse, calcium signaling, and muscle contraction. **(B)** The network of biological pathways associated with upregulated genes described interactions among pathways related to inflammatory response. **(C)** Schematic summary of the major transcriptomic alterations identified in the gastric antrum following loperamide treatment, including downregulation of synaptic transmission- and calcium signaling-related genes and upregulation of inflammatory response-related genes. The schematic represents transcriptomic associations and does not imply direct causal relationships among these pathways.

## 4 Discussion

Functional gastrointestinal disorders (FGIDs), including FD and constipation, affect a substantial proportion of the global population, and loperamide is frequently used to replicate these conditions in animal models owing to its potent anti-laxative effect in the GI tract (Jeon et al., 2019; Wang et al., 2023). FD, which has a global prevalence of 10–30%, is a disorder in which digestive function is critically dependent on the complex interactions between the gut and the nervous system; however, its etiology and pathophysiology remain poorly understood due to the multifactorial and psychosomatic nature of the condition (Holland et al., 2021). Although animal models for studying FD are not complete, researchers have frequently applied loperamide using mainly its ability to delay gastric emptying (**Figure 1B**). Gastric dysmotility is a predominant symptom in FD, corresponding to approximately 76% of FD, mainly related to types of postprandial distress syndrome (PDS) and their mixed type, except epigastric pain syndrome (EPS) (Asano et al., 2016). Although loperamide-induced delayed gastric emptying does not fully reproduce the multifactorial pathophysiology of FD, it remains one of the most widely used pharmacological models for studying impaired gastric motility.

Herein, we analyzed the transcriptional changes involved in loperamide-induced delay of gastric motility. In order to explore the precise mechanisms of the action of loperamide on GI motility in terms of overall gene profiles, we performed RNA-seq analysis using the antrum tissues of mice. As expected, loperamide treatment significantly altered gene expression patterns, as evidenced by the clearly separated clustering in the MDS plot using 1,356 DEGs composed of 553 up- and 803 down-regulated genes (**Figure 1C**).

GI motility including gastric emptying is controlled by nervous systems mainly composed of extrinsic (mainly parasympathetic and sympathetic nerves) and intrinsic (submucosal and myenteric plexuses), and various hormones such as ghrelin, substance P, and motilin, leading to finally modulate smooth muscle contraction (Spencer and Hu, 2020; Kim et al., 2021; Mori et al., 2022). The loperamide is known to regulate the release of various neurotransmitters, including acetylcholine and substance P (Shi et al., 2014; Pannemans and Corsetti, 2018). In particular, acetylcholine is a major excitatory neurotransmitter in GI peristalsis via the binding to acetylcholine receptors in various components of GI tract such as the vagus nerve, smooth muscle, and interstitial cells of Cajal (ICC), respectively (Yajima et al., 2011). Some preclinical studies have shown that the inactivation of muscarinic acetylcholine receptors inhibits the release of acetylcholine and subsequently suppresses GI motility (Mandl and Kiss, 2007; Sung et al., 2018). Our results found that loperamide notably suppressed the expression of genes related to “Neuronal system” and “Chemical synaptic transmission” (**Figure 1E**). We confirmed that loperamide significantly downregulates both acetylcholine receptors (muscarinic receptor Chrm2 2.2-fold and nicotinic receptor Chrna9 1.5-fold) and glutamate receptor (Grm7 2.0-fold) (**Figures 2A,C**). Chrm2 is one of the major muscarinic receptor subtypes expressed in gastrointestinal smooth muscle and contributes to cholinergic regulation of motility (Schledwitz et al., 2021). In M2R knockout mice, there was a 3-fold decrease in smooth muscle contraction of the GI tract under its agonist treatment (Kitazawa et al., 2007). In addition, the significant downregulated Grm7, a gene encoding glutamate metabotropic receptor 7, may contribute to impaired enteric neurotransmission associated with gastric dysmotility because this receptor mediates glutamate-derived excitation of the enteric nervous system (Kirchgessner, 2001). Our network analysis also showed the overview of downregulations of the synaptic pathways and muscle contraction (**Figure 3A**). The synaptic pathway closely involves the release of neurotransmitters, such as serotonin, dopamine, and acetylcholine, which play a critical role in regulating GI motility (Hansen, 2003).

As expected, loperamide treatment also notably regulates the gene expression in the calcium-related pathway, evidenced by significant downregulations of Cacng5, Cacna1e, and Cacna1a (**Figures 2A, C**). Calcium channel activation is a key step in the contraction of smooth muscle and the autonomic nerve system in the GI tract, promoting gastric emptying and intestinal transit (Perrino, 2016). Loperamide inhibits calmodulin, a key molecule involved in the contraction of smooth muscle in the GI tract, by blocking the Ca^2+^ flux of calcium channels (Kochegarov, 2002). In fact, Cacna1a, encoding the voltage-gated calcium channel, is known as a target gene of loperamide (Sudhof, 2012; Manickam and Ramasamy, 2021). Furthermore, the gene of glial fibrillary acidic protein (Gfap) was downregulated (3.4-fold) under loperamide treatment (**Supplementary Table 2**). Gfap encodes a filament protein playing a key role in the maturation of enteric glial cells, thus this was known as a marker of enteric glial (Grundmann et al., 2019). Enteric glial cells express the muscarinic acetylcholine receptor binding with acetylcholine from neighboring enteric neurons, then sequentially stimulates enteric glial cells to transduce the signal for smooth muscle contraction (Delvalle et al., 2018).

Among the most markedly downregulated transcripts were several keratin family genes, including Krt4 (163.0-fold), Krt5 (130.6-fold), Krt1 (64.2-fold), and Krt14 (55.3-fold) (**Figure 1D**; **Supplementary Table 2**). Keratins are intermediate filament proteins that contribute to epithelial structural integrity and differentiation (Salas et al., 2016). Therefore, the coordinated downregulation of multiple keratin-related genes may suggest alterations in epithelial-associated transcriptional programs in the gastric tissue following loperamide treatment. However, several of these transcripts showed relatively low absolute expression levels despite their large fold changes, and some keratin genes are not predominantly expressed in gastric epithelium. Thus, these findings should be interpreted cautiously and cannot by themselves establish impaired gastric epithelial homeostasis. The observed changes may reflect a true epithelial response, differences in tissue composition or sampling, or other biological or technical variability. Further histological or cell-specific analyses will be required to clarify the biological significance of these keratin-related transcriptional changes.

On the other hand, as a very intriguing finding, we noticed that loperamide treatment was associated with upregulation of inflammatory response-related transcriptional pathways, as evidenced by DEGs data (**Figures 1E**, **2B**). Low-grade gastroduodenal inflammation has been implicated in the pathogenesis of FD (Sayuk and Gyawali, 2020). Gastroduodenal inflammations, notably by eosinophil infiltrations, has been observed in up to 40% of patients with FD, which is at least partially corresponding mechanism for delayed gastric emptying (Matsumoto et al., 2008; Du et al., 2018). IL-33 has been implicated in immune activation and inflammation in patients with FD and IBS-constipation type (Andoh and Nishida, 2023), while our results showed the high expression of Il33 in loperamide-treated stomach tissue (2.4-fold in RNAseq **Figure 2B**, 2.2-fold in qPCR **Figure 2C**). The IL-33 is known to function as an inflammatory mediator recruiting the various immune cells, including eosinophils, in the peripheral and central nervous system (Simi et al., 2007). One possible interpretation is that delayed gastric emptying may promote local tissue stress and contribute to the induction of Il33-associated inflammatory responses (**Figure 3C**). The concomitant upregulation of interleukin 1 receptor-like 1 (Il1rl1, upregulated 4.0-fold) and Nuclear factor of kappa light polypeptide gene enhancer in B cells inhibitor, alpha (Nfkbia, 1.4-fold) may also be consistent with activation of inflammation-related signaling pathways; however, the present transcriptomic data do not establish activation of the IL-33/IL1RL1/NF-κB pathway at the functional level. Moreover, because histological inflammation or immune-cell infiltration was not directly evaluated, these findings should be interpreted as an inflammatory response-related transcriptional signature rather than direct evidence of gastric inflammation. Whether these transcriptomic alterations reflect resident-cell activation, immune-cell infiltration, or changes in tissue cellular composition remains to be determined.

In network analysis, upregulated inflammatory response was closely linked to leukocyte activation and adaptive immune response (**Figure 3B**). It is also noted that loperamide treatment significantly downregulated myosin heavy polypeptides (Myh) family genes, including Myh-10 and 11 (both 1.5-fold), which is a major component of smooth muscle and functions as a contractile protein in the GI tract (Wang et al., 2022). Some studies showed that gastroduodenal inflammation decreased the expression of smooth muscle heavy chain along with other contractile proteins like α-actin and C-kinase potentiated protein phosphatase-1 inhibitor (CPI-17) (Ohama et al., 2007).

To place our findings in context, we considered previous intestinal transcriptomic studies of loperamide-induced dysmotility (Xu et al., 2023; Chen et al., 2024; Sun et al., 2024). Several studies similarly reported enrichment of inflammatory response-related pathways and Il33 upregulation. However, direct comparison is limited by differences in species, gastrointestinal region, loperamide administration, and experimental design. Therefore, these observations should be regarded as qualitative cross-study similarities, and the present study does not establish gastric-specific transcriptional changes. Nevertheless, characterization of the gastric antrum remains relevant to the gastric dysmotility component of FD.

Our present findings enrich the knowledge to explain the detailed mechanisms of how loperamide modulates gastric emptying and GI motility, including loperamide-derived complications, in the clinic. This study has several limitations. Because the pharmacokinetic profile and tissue exposure to loperamide may differ according to the route of administration, the transcriptomic findings observed in the present study should be interpreted within the context of the intraperitoneal administration protocol used herein. In addition, transcriptomic profiling was performed using a single dose of loperamide at a single time point. Therefore, the present findings represent a snapshot of the transcriptional state associated with acute loperamide-induced gastric dysmotility and may not capture dose-dependent or temporal changes, including early transient and later adaptive responses. The transcriptomic analysis was performed only in male mice, which may limit the generalizability of the present findings. Sex-related differences in gastric motility and functional dyspepsia have been reported, including generally slower gastric emptying in women than in men (Kim and Kim, 2020; Narayanan et al., 2021). However, direct evidence regarding sex-dependent effects of loperamide on gastric motility remains limited. Therefore, whether the transcriptomic alterations observed in the present study are similarly reproduced in female mice remains to be determined. Because bulk RNA-seq was performed using whole gastric antrum tissues, the present data cannot distinguish cell-autonomous transcriptional changes within specific cell types from changes in tissue cellular composition. Therefore, the cellular origins of the observed DEGs remain unresolved, and further cell-specific, single-cell, or spatial analyses would be required to clarify these changes. Furthermore, the identified transcriptomic alterations were not validated at the protein or functional level. Additionally, a wash-out or recovery experiment was not performed; therefore, the reversibility and temporal persistence of the observed transcriptional alterations after withdrawal of loperamide remain unknown. Despite these limitations, our findings provide novel transcriptomic evidence suggesting that gastric inflammatory response-related transcriptional alterations, including Il-33-associated changes, may accompany loperamide-induced gastric dysmotility (**Figure 3C**).

Taken together, to our knowledge, this study provides the first transcriptomic characterization of loperamide-induced gastric dysmotility in gastric tissue. The present results will help us to comprehensively understand loperamide-derived pharmacological applications and drug-study animal models.

## Data Availability

The original contributions presented in the study are included in the article/**Supplementary Material**. Further inquiries can be directed to the corresponding authors.
