# Supplementary material for: Transcriptomic insights into loperamide-induced gastric dysmotility: RNA-seq analysis of a mouse model
Source: Front Physiol. 2026 Oct 7;17:1924065. doi: 10.3389/fphys.2026.1924065 (PMC13683198; doi:10.3389/fphys.2026.1924065)
Supplement: Supplementary file 1 [file Table4.docx]

Supplementary Table 4. Primer sequence for real-time quantitative PCR

| Gene | Sense (5' → 3') | Anti-sense (5' → 3') |
| --- | --- | --- |
| Chrm2 | CGGCTTTCTATCTGCCTGTC | GGCATGTTGTTGTTGTTTGG |
| Chrna9 | GGAACCAGGTGGACATATTCAAT | GCAGCCGTAGGAGATGACG |
| Grm7 | CCT GCC TGC TTC CTA TCTCTG | CCG GGT GGG ACT TGA ATC TC |
| Cacna1a | CGGAGCACAATAACTTCCGG | CGAGCACAGGAAGATGAACG |
| Il33 | ATGGGAAGAAGCTGATGGTG | CCGAGGACTTTTTGTGAAGG |
| Gapdh | AACAGGGTGGTGGACCTCAT | AGTTGGGATAGGGCCTCTCTT |

Chrm2, cholinergic receptor muscarinic 2; Chrna9, cholinergic receptor nicotinic alpha 9 subunit; Grm7, glutamate metabotropic receptor 7; Cacna1a, calcium voltage-gated channel subunit alpha1 A; Il33, interleukin 33; Gapdh, glyceraldehyde-3-phophate dehydrogenase.
